# Development of a Recognition System for Spraying Areas from Unmanned Aerial Vehicles Using a Machine Learning Approach

**DOI:** 10.3390/s19020313

**Published:** 2019-01-14

**Authors:** Pengbo Gao, Yan Zhang, Linhuan Zhang, Ryozo Noguchi, Tofael Ahamed

**Affiliations:** 1 Graduate School of Life and Environmental Sciences, University of Tsukuba, Tsukuba 305-8572, Japan; s1630258@u.tsukuba.ac.jp (P.G.); s1630260@u.tsukuba.ac.jp (Y.Z.); zhanglinhuanch@yahoo.co.jp (L.Z.); 2 Faculty of Life and Environmental Sciences, University of Tsukuba, 1-1-1 Tennodai, Tsukuba 305-8572, Japan; noguchi.ryozo.gm@u.tsukuba.ac.jp

**Keywords:** precision agriculture, recognition system, image classifiers, machine learning system, mutual subspace method

## Abstract

Unmanned aerial vehicle (UAV)-based spraying systems have recently become important for the precision application of pesticides, using machine learning approaches. Therefore, the objective of this research was to develop a machine learning system that has the advantages of high computational speed and good accuracy for recognizing spray and non-spray areas for UAV-based sprayers. A machine learning system was developed by using the mutual subspace method (MSM) for images collected from a UAV. Two target lands: agricultural croplands and orchard areas, were considered in building two classifiers for distinguishing spray and non-spray areas. The field experiments were conducted in target areas to train and test the system by using a commercial UAV (DJI Phantom 3 Pro) with an onboard 4K camera. The images were collected from low (5 m) and high (15 m) altitudes for croplands and orchards, respectively. The recognition system was divided into offline and online systems. In the offline recognition system, 74.4% accuracy was obtained for the classifiers in recognizing spray and non-spray areas for croplands. In the case of orchards, the average classifier recognition accuracy of spray and non-spray areas was 77%. On the other hand, the online recognition system performance had an average accuracy of 65.1% for croplands, and 75.1% for orchards. The computational time for the online recognition system was minimal, with an average of 0.0031 s for classifier recognition. The developed machine learning system had an average recognition accuracy of 70%, which can be implemented in an autonomous UAV spray system for recognizing spray and non-spray areas for real-time applications.

## 1. Introduction

With the development of unmanned aerial vehicle (UAV) technologies, the use of UAVs has rapidly expanded to different applications such as aerial photography to monitor vegetation, survey mapping, and scouting with wireless networking [1]. UAVs have the potential for use in agricultural applications, and they are ideal for precision agriculture, compared to aerial mapping and satellite remote sensing. The use of UAVs is not only more efficient, but also more cost-effective than areal or high-resolution commercial satellite datasets [1,2,3,4,5]. They can help monitor crops in real-time and provide high-resolution images of the field and canopy, for crop growth and production. High-resolution and machine vision images are used for the identification of weeds and non-weed areas, using ground-based conventional sprayers [6,7]. In recent advancements, sprayers have been attached to UAV systems to deliver sprays in the field. However, as the payload of a UAV with a sprayer makes it heavier, it becomes difficult to fly in the field while carrying large quantities of liquid chemicals. The process of spraying agricultural crops with liquids needs to be very efficient, to avoid spraying non-crop areas. Similarly, the orchard spray system needs to fly at a high altitude, to spray chemicals on the top of the canopy. High payloads of chemicals in the tank also cause problems. Large tank sizes require more power, and generate more safety concerns while flying. It is very important to recognize the spray areas above orchards and non-orchards, to ensure the precise application of spray chemicals. For the autonomy of UAV-based spraying systems, the ability to recognize crop and orchard areas is significantly important. Most autonomous and artificial intelligence systems need to be trained on data, prior to application. Training provides confidence in operations. Machine learning systems have the potential for training and testing on datasets to add artificial intelligence for different agricultural operations. In the spraying system, a machine learning system is required to discern spraying spots and non-spraying spots in operational environments of UAVs, prior to implementing an autonomous spraying system. Ground-based vehicles can function as image processing systems, with advantages for housing onboard sprayers [8,9]. However, ground vehicles have local mapping systems without predetermined field coverage. UAVs have the advantage of identifying the field coverage in advance, with good trained datasets and machine learning systems.

Most studies using UAV have employed limited dataset collection and machine learning systems. Some research has reported that aerial applications result in only 50% of the targets being sprayed from altitudes of less than 1 m [10]. To the best of our knowledge, UAV-based sprayers were introduced to the market, and largely implemented in mountains and cropping areas, to enable spraying with precision. Most commercial UAVs with sprayers are operated under regulations in many countries. As the technology is tending toward the development of autonomous systems, it is likely that UAV spray systems will have a high potential for autonomous spraying applications. To increase the flight time, UAV manufacturers have improved the endurance of systems by increasing the battery capacity, and reducing the total weight of the UAVs. In this regard, the application efficiency of chemicals to the spray area from a high altitude must be improved. The height of the operation greatly influences how orchard areas can be covered in a minimum amount of time. A similar consideration in selecting the height of the operation pertains to croplands. 

Unmanned aerial systems (UASs) have mounted sensors that offer an extraordinary opportunity for bridging the existing gap between field observations and traditional air and space-borne remote sensing, by providing high spatial detail over relatively large areas in a cost-effective way. UAV systems also have enhanced temporal retrieval for natural and agricultural ecosystem monitoring, in order to identify future directions, applications, developments, and challenges [3]. Small UAVs (approximately 1.5 kg) are capable of measuring the range to a water surface, using a global navigation satellite system (GNSS) receiver with radar, sonar, and an in-house-developed camera-based laser distance sensor (CLDS) for determining the water level (orthometric height) [4]. The remote detection of water stress in a citrus orchard was also investigated by using leaf-level measurements of chlorophyll fluorescence, and photochemical reflectance index (PRI) data, seasonal time-series of crown temperature and PRI, and high-resolution airborne imagery [5]. These studies have significant impacts on the application of UAV in agriculture and hydrology. Furthermore, machine learning systems have the opportunities for decision-making of remote sensing applications while using UAV-based sprayers.

How to generate quantitative remote sensing products by using rotating-wing and fixed-wing UAVs equipped with commercial off-the-shell (COTS) thermal and narrowband multispectral imaging sensors was also evaluated for vegetation monitoring. Radiometric calibration, atmospheric correction, and photogrammetric methods were employed, which are required to obtain accurate remote sensing products that are useful for vegetation monitoring [11]. The growing research community comprises tech-enthusiastic hydrologists that aim to design and develop their own sensing systems and to adopt a multi-disciplinary perspective to tackling complex observations, often using low-cost equipment that is intended for other applications to build innovative sensors for measurements [12]. A previously reported UAV system was integrated with optical sensing to allow for the quantitative characterization of surface flow phenomena, to yield accurate surface flow maps of sub-meter water bodies [13]. Vegetation monitoring and surface flow phenomena help in further confirmation of UAV applications for the recognition of classifiers. These studies significantly increase the scope of using UAV applications in the research community.

UAVs equipped with inexpensive thermal and narrowband multispectral imaging sensors have been used for agricultural applications, and have yielded comparable estimations [14]. Relying on UAV-based remote sensing and imaging techniques, high-throughput field phenotyping (HTFP) was conducted by using thermal imaging for the field phenomics of poplar and other tree species, for accelerating forest tree genetic improvement against abiotic stress [15]. HFTP and genetic improvement against abiotic stress also further increase applications of machine learning systems. Several machine learning systems have been introduced in ground-based sprayers using deep learning neural networks and Bayesian classifiers [16,17,18]. Most machine learning systems have high complexity data training and large time requirements for real-time applications. In our previous research, we found that the kernel mutual subspace method (KMSM) has a high potential for recognize features and actions of tracking, with accuracies of greater than 80% in real-time [19]. Furthermore, the KMSM, along with the Hankel matrix were used for action recognition of the machinery operator with a processing time of 0.07 s [19]. The mutual subspace method (MSM) has been used for the recognition of human faces and objects [20,21,22]. MSM has the advantage of object recognition from image sets or video sequences, due to receiving a variability of patterns, to achieve higher performances compared to other methods [22]. Therefore, the mutual subspace method has a highly promising capability in machine learning systems for recognizing features. In on-board spraying applications using UAVs, the recognition of features with minimum time and high accuracy can be performed using MSM. 

Therefore, the objective of this research is to develop a machine learning system for recognizing the features of spraying and non-spraying areas for applying UAV-based sprayers in agricultural croplands and orchards. It is expected that MSM machine learning systems can be employed, offering the advantages of low computational complexity and good accuracy in feature recognition systems for real-time applications.

## 2. Materials and Methods

### 2.1. Mutual Subspace Method (MSM)

**Figure 1:**
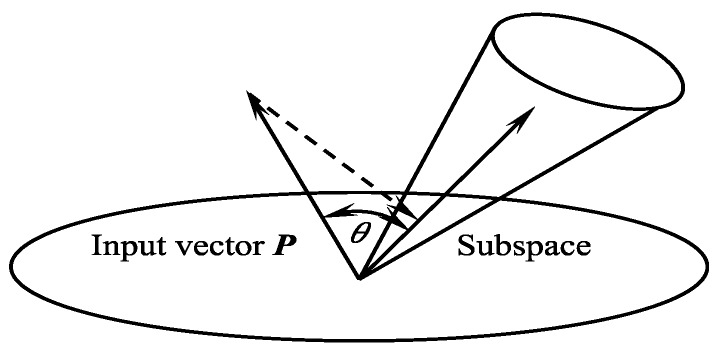
Subspace method (SM).

The MSM was introduced to the field of pattern recognition, a well-known method for object recognition based on image sets [23]. MSM is an extension of the subspace method (SM) that classifies a set of input pattern vectors into several classes, based on multiple canonical angles between the input subspace and the class subspaces (Figure 1). The input subspace was generated from a set of input patterns as a class [23,24]. The SM has a high performance in pattern recognition and was developed independently as CLAFIC (the class-SELFIC method: the original version of the subspace model) [25] and the multiple similarity method [26]. It classifies an input pattern vector into several classes, based on the minimum distance or angle between the input pattern vector and each class subspace, where a class subspace corresponds to the distribution of pattern vectors of the class in high-dimensional vector space [27].

We considered that the input vector ***P*** and *m* class subspaces belong to a *k*–dimensional vector space; the similarity is defined by the length or the minimum angle between the input vector ***P*** and the *i-*th class subspace, where the length of ***P*** is often normalized to 1.0. The angle-based similarity can be derived as follows:

(1) $$\cos^{2}\theta =\sum_{i=1}^{d}\frac{{\left(P\cdot \varphi_{i}\right)}^{2}}{\left\|P^{2}\right\|}$$

where *d* is the dimension of the class subspace, and $\varphi_{i}$ is the *i-th k*–dimensional orthogonal normal vector (PCA). First, the conventional PCA operates by diagonalizing the covariance matrix ***C***from *k* feature vectors $\vec{x_{j}}$ (a = 1, 2, …, k) in an n-dimensional feature space, $R^{n}$:

(2) $$C=\frac{1}{k}\sum_{j=1}^{k}\left(\vec{x_{j}}\cdot \vec{x_{j}}{}^{T}\right)$$

It gives an eigen decomposition of the covariance matrix by PCA to obtain the principal components $\vec{\nu_{i}}\left(i=1,\;2,\ldots ,k\right)$ of the distribution:

(3) $$\lambda \vec{\nu }=C\vec{\nu }$$

However, we assume that all of data here were calculated from the data centroid. This principal component describes the direction of the largest data variation under a linear approximation [23]. The above characteristic equation can be transformed as follows:

(4) $$\lambda \vec{x}=\left[\frac{1}{k}\sum_{j=1}^{k}\left(\vec{x_{j}}\cdot \vec{x_{j}}{}^{T}\right)\right]\vec{\nu }$$

(5) $$=\frac{1}{k}\sum_{j=1}^{k}\left(\vec{x_{j}}\cdot \vec{x_{j}}{}^{T}\right)\vec{\nu }=\frac{1}{k}\sum_{j=1}^{k}\vec{(x_{j}}\cdot \vec{\nu })\vec{x_{j}}$$

Because $\vec{\nu }$ is in $\left\{x_{1}\ldots ,\;x_{k}\;\right\}$, we obtain:

(6) $$\lambda \left(\vec{x_{a}}\cdot \vec{\nu }\right)=\vec{x_{a}}\cdot C\vec{\nu }$$

where $\vec{x_{a}}$ is a feature vector.

**Figure 2:**
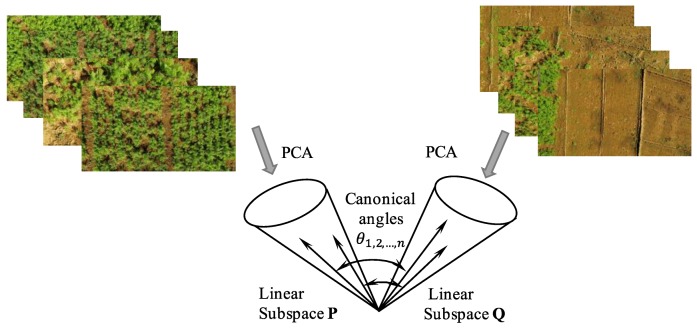
Comparison between two sets of images using the mutual subspace method (MSM).

The MSM has been used to compare small variations in the training data and recognition target data, and this results in a powerful recognition technique when the data distribution can be linearly approximated, which occurs when multiple data can be used as recognition target image inputs. In the subspace method, a subspace that has d-dimensional vectors is selected according to a criterion such as the cumulative contribution rate from the eigenvectors, which are obtained by using PCA on the entered images [22,26,28]. Then, the similarity between the subspaces is defined according to the angle θ between the eigenvectors $P=\left\{\vec{\mu_{i}}\right\}$ (registered as a dictionary) and the eigenvectors $Q=\left\{\vec{\nu_{j}}\right\}$ (obtained from the input data) (Figure 2).

According to Equation (1), the angle θ between the subspaces is given as the maximum eigenvalue [21,22]:

(7) $$\cos\theta =\underset{\vec{\mu_{i}}\in P}{\max}\;\underset{\vec{\nu_{i}}\in Q}{\max}{\vec{µ}}^{T}\vec{\nu }$$

where ${\vec{\mu_{i}}}^{T}\;\vec{\mu_{i}}={\vec{\nu_{j}}}^{T}\;\vec{\nu_{j}}=1$, ${\vec{\mu_{i}}}^{T}\;\vec{{µ}_{j}}={\vec{\nu_{i}}}^{T}\;\vec{\nu_{j}}=0$, i≠j, 0<i,j≤d, and d is the dimensionality of the subspace used for recognition.

### 2.2. Research Design for Classifiers and MSM 

**Figure 3:**
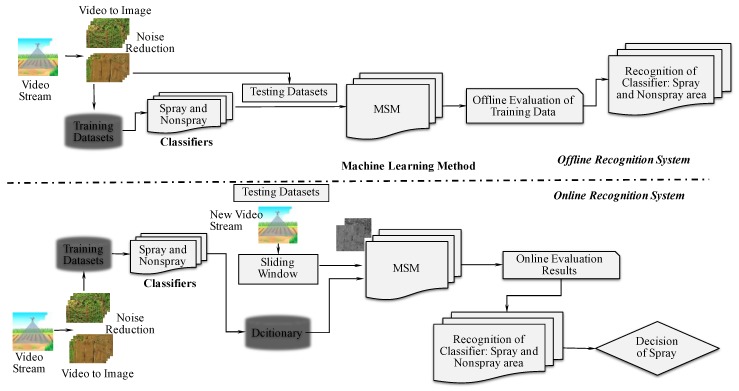
The research framework establishing the classifiers and the MSM.

The classifiers are required to be established before the MSM application. The MSM research approach involves two steps: offline and online recognition systems. The offline recognition system was used to validate the model and the accuracy of the recognition of the classifiers (Figure 3). The online recognition system was proposed, to determine the computational times required to enable the real-time system. In offline recognition, videos must be captured by using the UAV, and converted through a JPG converter. For offline recognition, selected images were taken from different datasets of crops and orchards for training and testing of the classifiers. For online recognition, a new video stream was captured. From the stream video, one frame was chosen out of 20 frames from a new video stream. Considering the restricted computational time required by a real-time system, RGB images were converted to grayscale. While testing using the online recognition system, a sliding window was used to obtain four consecutive frames, and PCA was applied, using the subspace method. In the subspace method, multiple images were required, and we noted that four frames were optimal for use in the subspace method.

In the following sections, details of field experiments for training and testing different datasets and the offline and online recognition systems are described.

### 2.3. Field Experiment for Training and Testing with Datasets

**Figure 4:**
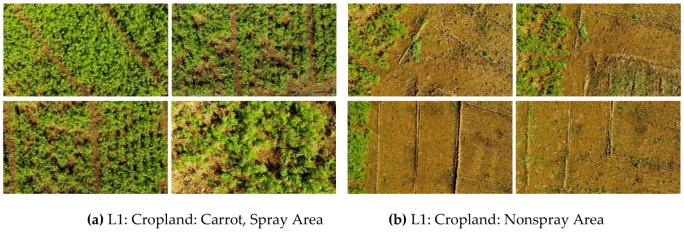

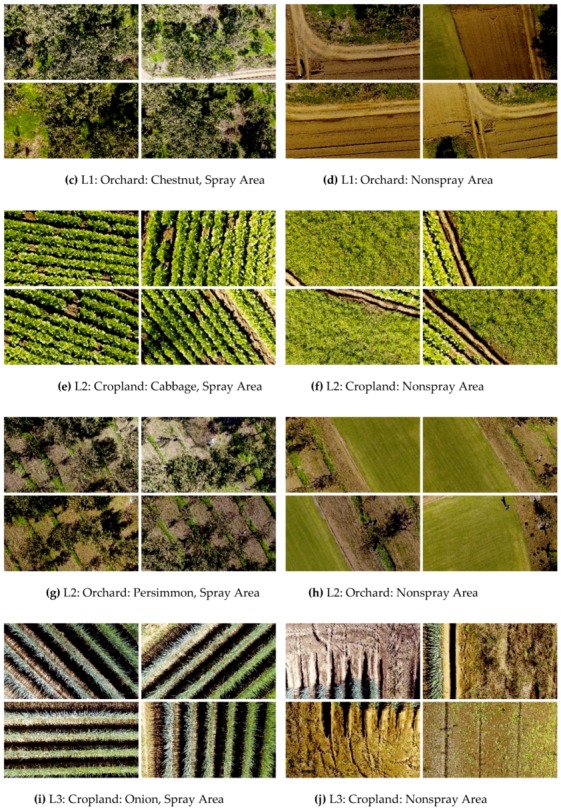

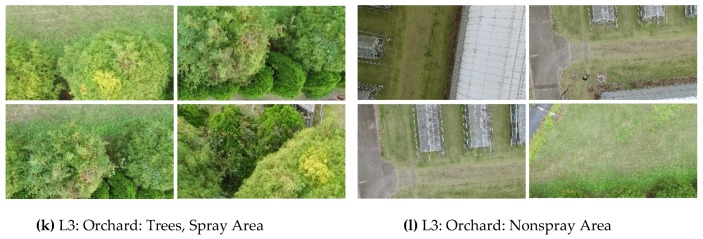
**(a–l)** Training and testing datasets for building the classifiers for recognizing spray areas and non-spray areas.

To implement the MSM for feature recognition, different crops and orchards are required for training the subspace patterns and verifying the recognition accuracy. While selecting the datasets for training, image acquisition at close range was preferable for agricultural croplands. On the other hand, for orchards, a high altitude allowed the canopy to be covered in a minimum time. Generally, close-range spraying can effectively reduce the drift and waste of chemicals. However, the UAV sprayer payload and the battery operational time are major concerns in enabling autonomous spraying. In this study, two working patterns were defined, depending on the flying height. The corresponding work areas are described as follows: for cropland (i.e., carrot, cabbage, and onions), the plant height was less than 5 m, and image acquisition was performed by using a UAV from a height of 5 m. In the case of orchards or plantations (i.e., chestnut, persimmons, and tall trees), we considered the height of the orchards to be less than 15 meters, and thus, the acquisition of images was conducted from a height of 15 m from the ground (Table 1). Two classifier datasets were collected for cropland spray area recognition: one dataset for spray areas (carrot, cabbage, onions) and another dataset for non-spray areas (inner farm roads, ridges, bare soil). Similarly, two classifier datasets (spray and non-spray areas) were also collected for orchards: one dataset for orchard areas (chestnuts and persimmon), and another dataset for trees, which included structured areas (farm houses, green house structure, farm buildings). The classifier datasets were captured by using a commercial UAV (DJI Phantom 3 Pro) with an onboard 4K camera with 1/2.3″ CMOS and FOV 94° 20 mm f/2.8 lens. The 4K videos were collected and converted to images by using a JPG converter at the preprocessing stage. The images were collected in the morning from 10 a.m. to 12 p.m., to ensure uniform lighting while the UAV flew over the croplands and orchards. Days with clear skies were generally chosen for collecting the videos by flying the UAV. The classifiers were segmented from the videos according to the flight heights for croplands and orchards (Table 1). Three field experiments were conducted with the UAV in three randomly selected zones; a rural farm with a combination of croplands and orchards (L1), a farm with different croplands with orchards (L2), and a research farm with croplands and orchards (L3) (Figure 4a-l). MATLAB 2015a^®^ (MathWorks, MA, USA) was used to develop the user interface and training and testing datasets for offline and online recognition systems.

### 2.4. Offline Recognition System

**Figure 5:**
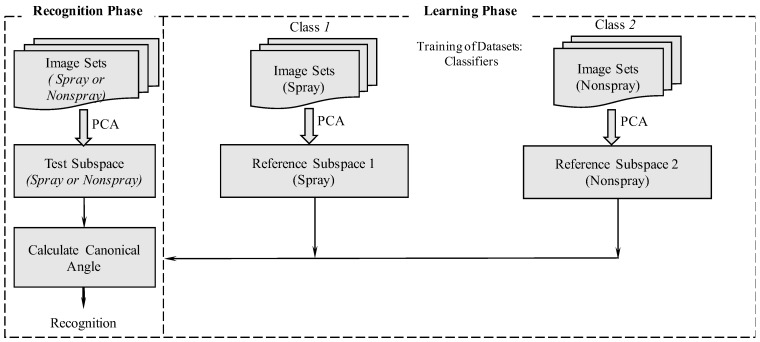
Image sets in classifier recognition in the learning and recognition phases for MSM applications.

The offline recognition system consisted of learning and recognition phases. The learning phase was commenced by collecting training image datasets of each class m∈{1,…,M}, and inputting them into the system. For offline experiments of each land type, we used one of the videos, with the first half for training, and the last half for testing. The recognition phase was confirmed to begin, once the learning phases of the classifiers using scene sequences were completed (Figure 5). Then, PCA was applied, to establish the linear subspace as a reference subspace for each class. The training phase was completed in three stages. First, all of the collected testing images of $I_{j}\in \left\{1,\ldots ,J\right\}$ were input into the system, and each *I* had frames of $\left\{f_{1},\ldots ,f_{n}\right\}$. Second, PCA was applied to establish the linear subspace for testing the subspace for each class $I_{j}$. Finally, the canonical angles between the current testing subspace and each reference subspace were calculated. The current image was assigned to the class with whom it shared the smallest canonical angles, which indicated that it had the highest similarity when referenced to the training datasets. In an offline experiment setting, the UAV was flown 5 m above the cropland. The first half of the images for training (99 images, spray, and 99 images, non-spray) and the last half of images for testing (99 images, spray, and 99 images, non-spray) were selected for cabbage fields (Table 1). The first half of the images for training (53 images, spray, and 53 images, non-spray) and the last half of the images for testing (54 images, spray, and 54 images, non-spray) were selected for onion fields. Similarly, the first half of the images for training (60 images, spray, and 60 images, non-spray) and the last half of the images (60 images, spray, and 60 images, non-spray) were selected for testing carrot fields. A height of 15 m was chosen for flying over orchard areas to collect the first half of the images for training (48 images, spray, and 48 images, non-spray) and the last half of the images for testing (49 images, spray, and 49 images, non-spray) for chestnut trees. Again, the first half of the images for training (47 images, spray, and 47 images, non-spray) and the last half of the images for testing (47 images, spray, and 47 images, non-spray) were used in the case of the persimmon fields. Finally, the first half of the images for training (59 images, spray, and 59 images, non-spray) and the last half of the images were used for testing (59 images, spray, and 59 images, non-spray) for trees and structures. The accuracy analysis of the offline recognition system was compared with the true positive and true negative values (Table 2). For further confirmation, the extended datasets were considered, to check the recognition accuracy of classifiers by using MSM.

### 2.5. Online Recognition System

**Figure 6:**
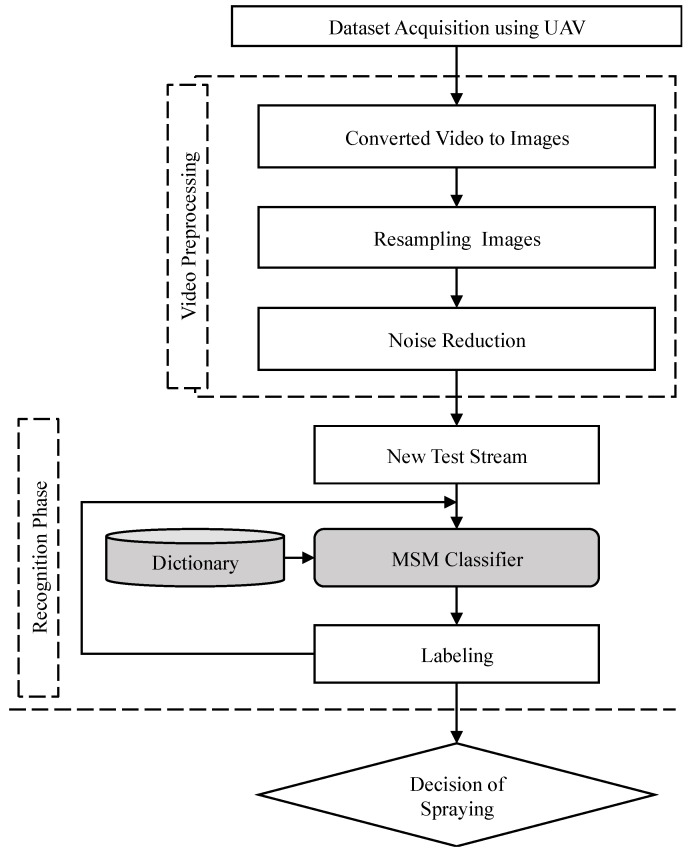
Online recognition system for the classification of spraying, based on MSM classifiers.

The subspace patterns were trained during the offline recognition process. These patterns were used for the online recognition development of classifiers. A sliding window was used to select four images that were converted to four vectors through resizing and reshaping. The grayscale images were resized to 8 by 8 and reshaped to one column vector using MATLAB^®^. A test subspace was generated using PCA for creating a matrix from the vectors. The online recognition progress was completed in the following stages. First, each video from each target crop or orchard was preprocessed, and one image was extracted from every 20 frames. Among the extracted images, there were several frames captured that did not belong to either class during takeoff and landing, or that included other plants during entry and exit. Such images were marked as noise images and removed to improve recognition accuracy. In the experiment, two datasets were collected for each target land. For the online experiment, we used all of the frames (removed noise) from one of the videos as training, and we used another video for testing (the video was not directly read; rather, the video was extracted to image frames, and the noise was removed). In the second step, we classified the set of sequential images, using the MSM classifier. Finally, the spray areas were recognized, based on the training datasets (Figure 6). In the datasets, 198 images (spray) and 198 images (non-spray) were collected from a 5 m height for training, and a reference subspace was built for use in the online experiment for cabbage. In the case of testing, a new video was taken, where one frame was selected out of 20 frames. There were a total of 298 frames that were used for testing for cabbage. Similarly, 107 images (spray) and 107 images (non-spray) were selected for training in online experiments. The new video stream was used with a total of 204 images for onion. In the case of carrots, 120 images (spray) and 120 images (non-spray) were used for training, and a new video stream with 89 images was used for testing the datasets. For orchard categories from a height of 15 m, two classifiers were trained using 97 images (spray) and 97 images (non-spray); 94 images (spray) and 94 images (non-spray); 118 images (spray) and 118 images (non-spray) for chestnut, persimmon, and trees, respectively. For testing the datasets of each target, a new video stream was taken, with a total of 180 images being extracted for chestnut, 210 images for persimmon, and 141 images for trees.

## 3. Results

### 3.1. Offline Recognition Performance

In the offline recognition system, the accuracy was 80.5% in the cropland classifiers for spray and non-spray area recognition in the first experimental areas (L1). In the case of orchards, the spray and non-spray area recognition was 75% (Table 3). In the second experimental area (L2), the recognition accuracy was 70.4% and 86.1% for croplands and orchards, respectively. Finally, mixed crop and orchard areas (L3) were chosen for offline recognition by classifiers. The recognition accuracy was 72.3% and 70% for croplands and orchards, respectively. The overall accuracy was 74.3% (croplands) and 77% (orchards) for the L1, L2, and L3 locations, which had a combination of croplands and orchards (Table 3). Wide crop canopy or orchards had the advantage of higher recognition by classifiers. The high accuracy of the recognition system was obtained by using the MSM for training and testing the datasets from the three different types of experimental fields.

For further confirmation, frame numbers were increased for the training and testing of datasets, whether there were significant differences in the recognition accuracy of the classifiers. Extended datasets confirmed that the accuracy of MSM method did not change much, even if the frames were increased to double for the testing and training of datasets in the offline recognition system (Table 4). 

### 3.2. Online Recognition Performance

**Figure 7:**
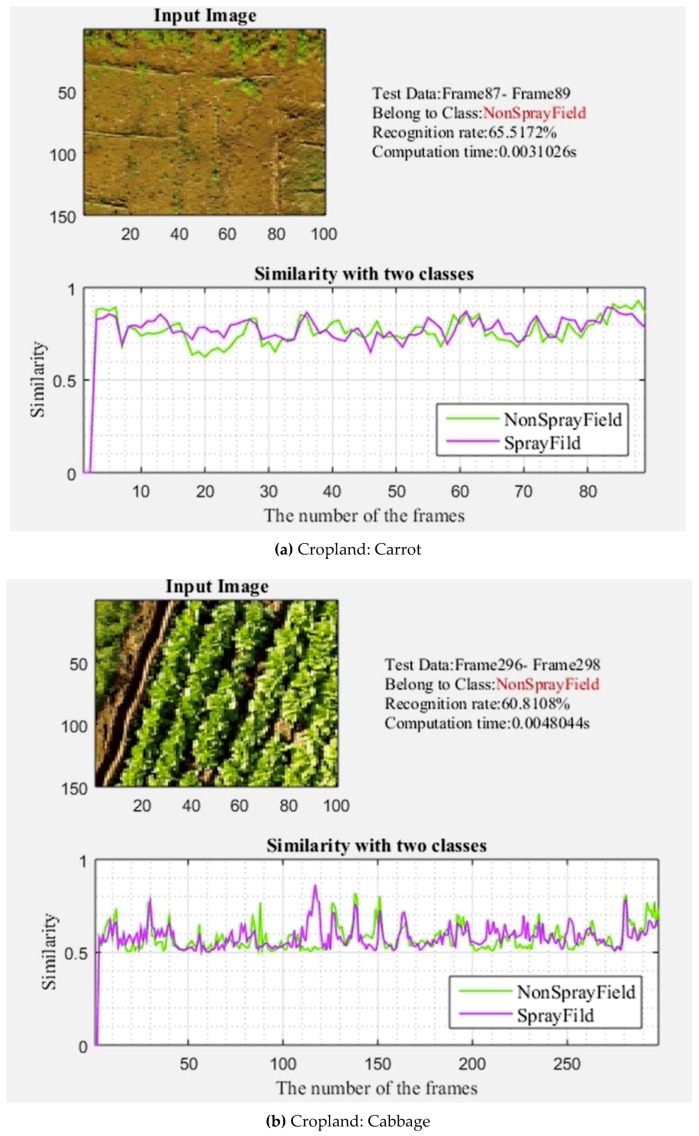

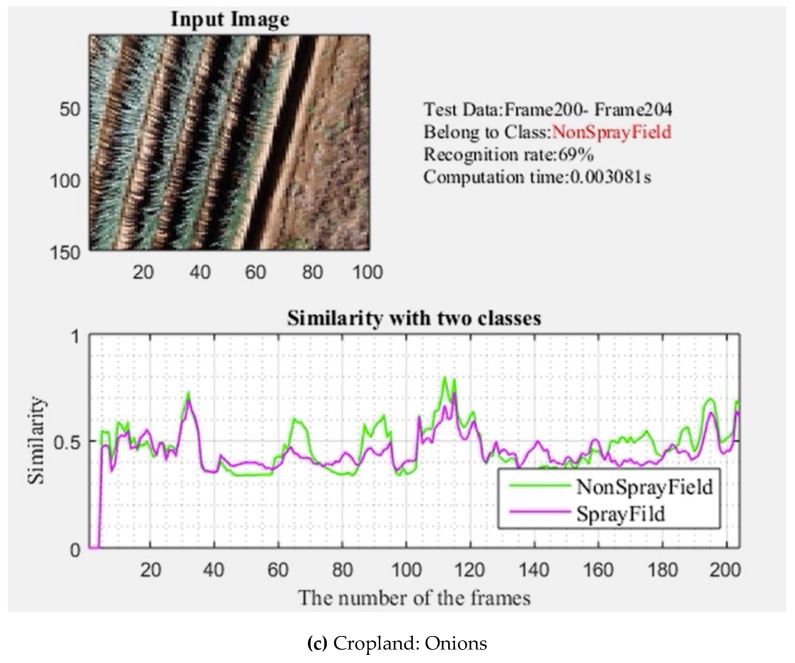
**(a–c)** Online recognition performance of a classifier of croplands from a height of 5 m.

**Figure 8:**
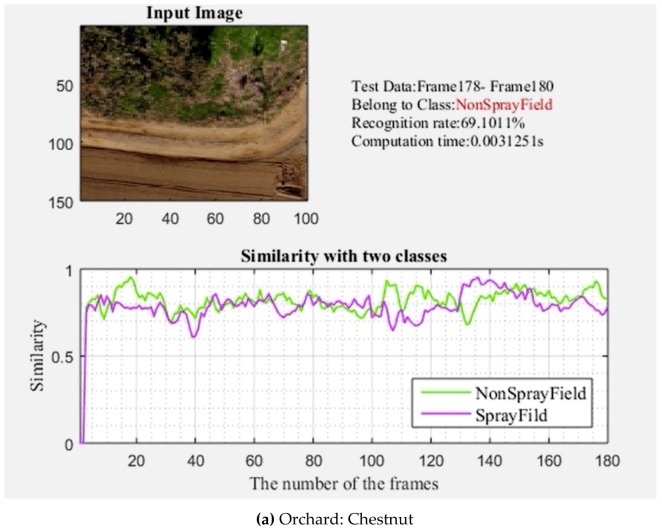

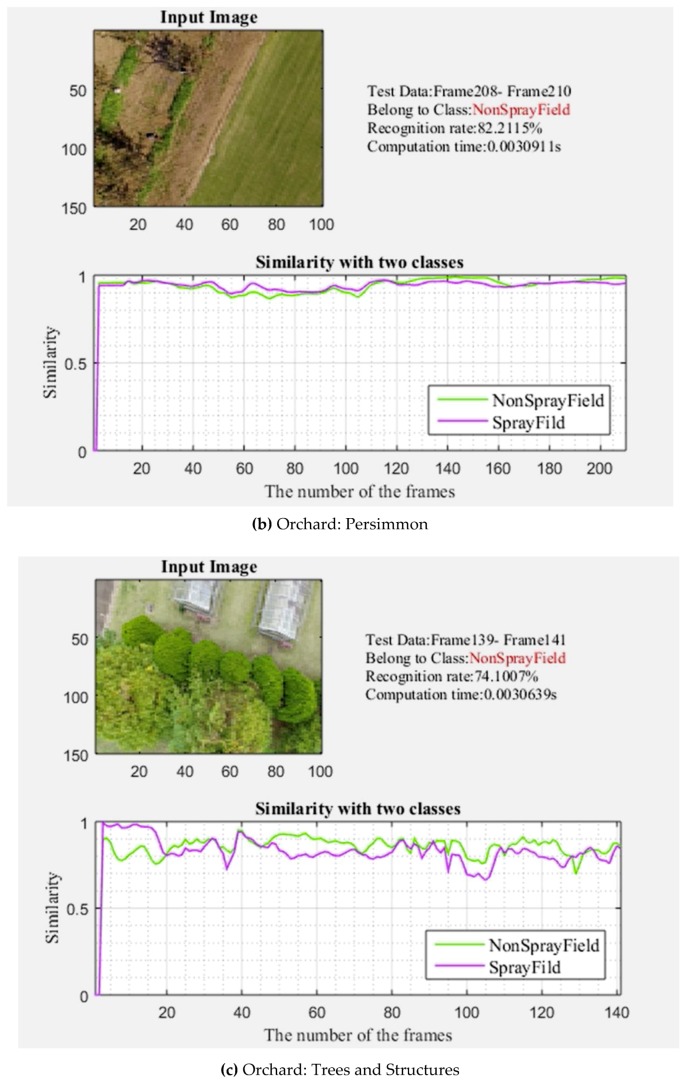
**(a–c)** Online recognition performance of a classifier of orchards from a height of 15 m.

The developed user interface had the advantage of online information, which included the current cropped image, the tested image sets using a sliding window, the predicted category, the recognition rate (the correct classifications were known during the test), the computational time, and the similarity plot. For the cropland classifiers, the UAV was flown at a 5 m height, and the recognition rate was observed to be 65.5% for the L1 experimental areas. The computational time was only 0.0031 s for classifier recognition (Figure 7a–c). The flying height was 15 m for orchard classification, and recognition was observed at 69.1%, with a computation time of 0.0031 s for each classifier. In the second experimental flying areas (L2), the recognition accuracy of the classifiers for the noted spray and non-spray areas was 60.8% and 82.2% for croplands and orchards, respectively. The computational time was only 0.0031 s for recognition by the classifiers, and orchard classifier recognition also required only 0.0031 s for each classifier (Figure 8a–c). In the third experimental location (L3), the online recognition rate by the classifiers reached 69% in 0.0048 s for each classifier, and 71.7% in 0.0031 s for each classifier, in croplands and orchards, respectively. The online recognition system had an average accuracy of 65.1% and 75.1% for croplands and orchards, respectively, with a recognition time of 0.0031 s (Table 5).

## 4. Discussion

The field experiments were conducted in different types of fields, to increase the dataset variety for the selection of spray and non-spray areas inside the croplands and orchards. The offline recognition system shows the effectiveness of MSM for training and testing of the datasets for croplands and orchards. The classifiers were used for croplands and orchards, and they were limited to being trained and tested on datasets that were acquired in the late fall season. MSM has the flexibility of increasing the number of classifiers, which may increase the computational time requirement. As UAV spraying is performed at higher speeds, we tend to focus on minimizing the computation time, to reduce the computational burden for decision-making, to recognize the spray and non-spray areas in croplands and orchards. UAVs operating at a high speed with limited battery life and a small payload of liquid chemicals demand high computational speeds and fast operation with good recognition accuracy. With this consideration, the online recognition system provided some advantages, although its accuracy was not as high as that of the offline recognition system. The system needs further training data to increase its accuracy, especially for the identification of croplands less than 5 m high and orchard areas from 15 m high. In the online experiment setting, similar environments resulted in increased recognition, while adding different categories of orchards reduced recognition. It was very challenging to test the datasets from a UAV fast operating speed at a high altitude. Classifiers were trained and tested on datasets acquired from three different locations to confirm the recognition accuracy. However, complex canopy systems were not present in the features. This MSM system had limitations in recognizing classifiers in complex canopies of crops or orchards. We could not collect images of complex canopy crops, and we assume that in such canopy systems, upward and downward image acquisitions are required to identify the spray and non-spray areas under different lighting conditions. Lighting is a key point that needs to be carefully considered, especially for its interception through the canopy. It would be ideal to train the UAV features of spray and non-spray areas on a large field, to obtain higher accuracies in precision applications ranging from normal to complex canopies of crops. Further studies are required to deal with such complexities of canopies, very large datasets in different lighting conditions, and the processing of images to remove noise by using extended Kalman filters, in onboard UAV systems. 

## 5. Conclusions

A machine learning system was developed by using MSM for images collected by a UAV in different types of farm fields and orchards. The machine learning system was developed to train and test two classifiers, one for agricultural croplands and one for orchard areas, on different datasets, to distinguish spray and non-spray areas for the future development of autonomous spraying systems. Images were collected from low (5 m) and high altitudes (15 m). The accuracy of the offline recognition system was found to be 74.4% and 77% for low- and high-altitude systems, respectively. On the other hand, the online recognition system performance had an average accuracy of 65.1% and 75.1% for low-altitude and high-altitude image acquisition systems, respectively. The computation time for the online recognition systems was observed to have a minimum of 0.0031 s (on average) for reporting classifier recognition. The developed machine learning system for recognizing the classifiers of spray and non-spray areas can be implemented in the autonomous UAV spray system in real-time. In our future experiments, we will improve the training and testing system by incorporating an artificial neural network (ANN) and deep learning to develop a UAV-based autonomous spraying unit for croplands and orchards.

## Figures and Tables

**Table 1:** Training and testing with datasets classified into two categories for offline and online recognition systems.

Targets	Data Sets		Training Image Numbers		Testing Image Numbers	
	Spray	Nonspray	Offline (Spray + Nonspray)	Online (Spray + Nonspray)	Offline (Spray + Nonspray)	Online
Carrot	120	120	First half (60 + 60)	All (120 + 120)	Last half (60 + 60)	New video (89)
Cabbage	198	198	First half (99 + 99)	All (198 + 198)	Last half (99 + 99)	New video (298)
Onion	107	107	First half (53 + 53)	All (107 + 107)	Last half (54 + 54)	New video (204)
Chestnut	97	97	First half (48 + 48)	All (97 + 97)	Last half (49 + 49)	New video (180)
Persimmon	94	94	First half (47 + 47)	All (94 + 94)	Last half (47 + 47)	New video (210)
Trees and Structures	118	118	First half (59 + 59)	All (118 + 118)	Last half (59 + 59)	New video (141)

**Table 2:** Accuracy analysis for the offline recognition system.

		True condition (offline recognition)		
		Spray	Nonspray	∑Total
Predicted condition (tested by recognition phase)	Spray	True Positive	False Positive	Total Positive
	Nonspray	False Negative	True Negative	Total Negative
	Accuracy	$Accuracy=\frac{\sum True\;Positive+\sum True\;Negative}{\sum Total}$		

**Table 3:** Offline classifier recognition and accuracy analysis.

			True condition (offline recognition)			
	Location (Croplands, Orchards)	Work patterns	Cropland		Orchard	
		Classifiers	Spray	Nonspray	Spray	Nonspray
Predicted condition (Tested by the recognition phase)	L1	Spray	74	21	35	9
		Nonspray	16	79	13	31
		Accuracy	80.5%		75%	
	L2	Spray	38	11	41	2
		Nonspray	18	31	10	33
		Accuracy	70.4%		86.1%	
	L3	Spray	56	0	37	18
		Nonspray	31	25	15	40
		Accuracy	72.3%		70%	

(L1: a farm with a combination of croplands and orchards, L2: a farm with different croplands with orchards, L3: a research farm with croplands and orchards)

**Table 4:** Extended datasets for the training and testing of classifiers, using an offline recognition system.

Croplands and Orchards	Data Sets		Training Image Numbers	Testing Image Numbers	Accuracy
	Spray	Nonspray	Offline	Offline	
Carrot	256	256	First half (128 + 128)	Last half (128 + 128)	73.79%
Cabbage	440	440	First half (220 + 220)	Last half (220 + 220)	81.25%
Onion	210	210	First half (105 + 105)	Last half (105 + 105)	66.32%
Chestnut	224	224	First half (112 + 112)	Last half (112 + 112)	77.31%
Persimmon	248	248	First half (124 + 124)	Last half (124 + 124)	70.94%
Trees and Structures	216	216	First half (108 + 108)	Last half (108 + 108)	64.58%

**Table 5:** Online classifier recognition and accuracy analysis

Croplands and Orchards	Flying Height (m)	Accuracy (%)	Recognition Time of Classifier (s)
Carrot	5	65.51	0.0031
Cabbage	5	60.88	0.0048
Onion	5	69.00	0.0031
Chestnut	15	69.10	0.0031
Persimmon	15	82.21	0.0031
Trees and Structures	15	74.10	0.0031
